# Tracking the dynamics of individual gut microbiome of sea cucumber *Apostichopus japonicus* during gut regeneration

**DOI:** 10.7717/peerj.10260

**Published:** 2020-12-01

**Authors:** Yohei Yamazaki, Yuichi Sakai, Juanwen Yu, Sayaka Mino, Tomoo Sawabe

**Affiliations:** 1 Laboratory of Microbiology, Faculty of Fisheries Sciences, Hokkaido University, Hakodate, Japan; 2 Hakodate Fisheries Research, Hokkaido Research Organization, Hakodate, Japan

**Keywords:** Gut microbiome, Gut regeneration, Sea cucumber, *Apostichopus japonicus*

## Abstract

Sea cucumbers possess the remarkable capacity to regenerate their body parts or organs. Regeneration of host organs and/or body parts involves reconstruction of the host associated microbiota, however, the dynamics and contribution of microbiota to the regeneration process are largely unknown due to a lack of experimental models. To track the dynamics of individual gut microbiomes during gut regeneration, both caged mariculture and laboratory isolator systems of sea cucumbers (*Apostichopus japonicus*) were developed and longitudinal meta16S analyses were performed. Under natural environmental conditions in the caged mariculture system, both bacterial and eukaryotic communities in sea cucumbers’ guts appeared to be reconstructed within 4 months after evisceration. Using the laboratory isolator, which can trace daily dynamics, we found that fecal microbiota collected before evisceration were clearly different from those collected after evisceration. We also identified eight key bacteria, belonging to Alteromonadaceae, Rhodobacteraceae, Oceanospirillaceae and family-unassigned Gammaproteobacteria, suggesting that these bacteria might interact with the host during the gut regeneration process. Six of the eight key bacteria were isolated for further bioassay using the isolator developed in this study to test whether these isolates affect gut regeneration.

## Introduction

Regeneration biology is one of the fundamental fields in biology (Nachtrab & Poss, 2012). Remarkable regenerative capacities have been observed in a wide range of animals species including mammals (e.g., African spiny mouse), amphibians (e.g., newts), urochordates (e.g., sea squirt), echinoderms (e.g., sea stars, sea cucumbers) and platyhelminthes (e.g., planaria) (Mashanov & García-Arrarás, 2011; Seifert et al., 2012; Umesono et al., 2013; Jeffery, 2015; Tanaka et al., 2016). As regeneration biology in the above animals has undergone research, a view has emerged splitting the continuum into three groups: wound healing, tissue repair, and regeneration (Galliot et al., 2017). Wound healing is often replaced by fibrotic scarring in mammals, and tissue repair involves functional restoration of the injured organs with no patterned 3D reconstruction. Regeneration involves regrowth and 3D patterning of complex structures, such as appendages or body parts, and is reliant on blastema formation (Galliot et al., 2017).

We readily expect that all regeneration processes could involve reconstruction of host associated microbiota, however, compared to the regeneration biology of higher organisms, studies on the regeneration of microbiome along with regeneration of host organisms are relatively new and emerging fields. As most animals are associated with microbes, a study on the regeneration of microbiome along with host animal regeneration has become an essential field in biology. Even so, until *Akkermansia*-enriched microbial communities were found to colonize colon wound beds in mice as a response to high anaerobic conditions and contribute to the healing of wounds (Alam et al., 2016), our knowledge of such host-microbe association in the regeneration process has been extremely limited.

In aquatic animals, studies on microbial dynamics during regeneration have also been undertaken recently using sea cucumber as an excellent regeneration model of both host and its associated microbiome. This helps us gain insight towards further understanding of “holobiont regeneration dynamics”. The animals show regrowth with a 3D patterning of the gut and respiratory tree in a short time following regeneration of its microbiomes on the complex re-structured substrata. Wang et al. (2018) first reported that the bacterial community structures and gut microbiome function of sea cucumber *Apostichopus japonicus* varied in different stages of gut regeneration using pooled samples of animals taken at various regeneration time points whilst being reared in an indoor facility (Wang et al., 2018). Zhang et al. (2019) further advanced studies on gut microbiome regeneration using three animals at each regeneration time point in a coastal rearing pond but animals were still dissected when analyzing the gut microbiome. They also supported the results of Wang et al. (2018) showing (1) the abundance of Bacteroidetes and Rhodobacterales (Proteobacteria) significantly increased in the gut microbiota of *A. japonicus* during the regeneration process, (2) the gut microbiota varied according to the progress of gut regeneration and finally restored similar microbiota to that before the loss of guts, and (3) gene frequencies facilitating cell proliferation, digestion and immunity increased in the microbiome of regenerating intestines, using both meta16S and metagenomic sequencing analyses (Zhang et al., 2019). More recently, Weigel (2020) analyzed gut microbiome dynamics of the sea cucumber *Sclerodactyla briareus* during gut regeneration in a compartmented aquarium in order to prevent cross-contamination of microbiomes, and concluded the gut microbes of the sea cucumber species were deterministically assembled, which could support previous observations using *A. japonicus*. However, all studies used dissection of individual sea cucumber to study the gut microbiome dynamics, no studies have tracked the gut microbiome dynamics using the same specific individuals during the whole process of gut regeneration not only in the open ocean but also in isolated aquariums.

Individuality and resilience are fundamental characteristics in maintaining beneficial gut microbial communities in humans (Sommer et al., 2017). Tracking the individuality and resilience of human gut microbiomes and/or those of other terrestrial animals after external pertubations (e.g., antibiotics) have been established in monitoring feces microbiomes, but there have been few studies analyzing those of aquatic animals including sea cucumber due to the difficulties of taking fresh feces samples under-water and to purify the DNA of small animals including juveniles. A recently developed approach designed “individual microbiome analysis” taking feces sample of cultured sea cucumbers on-site without sacrificing animals reveals (1) significant abundance of specific taxa belonging to Rhodobacterales possessing PHB metabolism in larger individuals of *A. japonicus* (Yamazaki et al., 2016), and (2) continuous selective enrichments of ingested microbes from environmental sediments as a driving force in shaping the gut microbiomes of wild sea cucumbers in Japan (Yamazaki et al., 2019). Using the individual microbiome approach, new insights into the structure and functional dynamics of sea cucumber gut microbiomes have quickly come to light.

Without tracking microbial community and those functional changes in the same specific individual, individuality and resilience of gut microbiome could not be properly assessed. Accelerating the related fields in the study of sea cucumber microbiome dynamics, we tried to apply the individual microbiome approach to help understand gut microbial community dynamics during gut-regeneration in both wild and laboratory fed animals of *A. japonicus*. Here, we demonstrated the caged mariculture and laboratory isolator system of sea cucumbers to track the dynamics of gut microbiomes derived from specific individuals during gut regeneration, thereby performing longitudinal meta16S analyses of the fecal microbial communities from both regenerated and control individuals. These studies support the resilience and individuality of the gut microbiome of *A. japonicus*, but the dynamics varied between individuals.

## Materials and Methods

### Caged mariculture system and sample collection at Menagawa site

The caged mariculture experiment was performed in a mariculture pond at the Menagawa site (41°45N, 141°5E), Hokkaido, Japan in December 2016. The size of the pond is 55 × 46 × 4 m (in height, width and depth), where fish and benthic organisms (e.g., sea cucumbers, sea urchins, sea stars, abalones, and algae classified to *S. japonica* and *Ulva* spp.) were co-present. Natural seawater was exchanged through open flow paths which connected the pond to the Ocean. The bottom of the pond is sandy and rocky, and thus sea cucumbers feed mainly on sands and surface biofilms and biomaterials covering the rocks.

We collected feces from seven wild individuals of the sea cucumber *A. japonicus* in December 2016. The body weight of individuals used in this study were 41–390 g, but the ages of the individuals could not be assessed due to lack of methodology. Fecal samples were immediately frozen using dry-ice and preserved at −80 °C in our laboratory before DNA extraction and purification. Using three individuals, we then mechanically removed the guts of sea cucumbers using sterilized wood sticks, because chemical treatments are prohibited in the mariculture pond. These three animals and another four were separated into two groups “gut-regenerated” and “control” groups, respectively, then placed individually into labeled cages set on the sea floor to track each specimen. We resumed collecting feces 4 months after the evisceration treatments in April 2017; one sample from the gut-regenerated group and three samples from the control group were collected. After this, feces were collected once a month; three (regeneration) and three (control) samples in May; two (regeneration) and three (control) samples in June; three (regeneration) and four (control) samples in July (Fig. S1). We could not take feces from some individuals due to lack of defecation. Samples were immediately frozen in dry-ice and preserved at −80 °C in our laboratory before DNA extraction and purification.

We did not need special permission in performing the experiments because the sea cucumbers were not caught or dissected.

### Laboratory isolator system and sample collection

Five specimens of *A. japonicus* in the regeneration group and three specimens in the control group were used. Each sea cucumber was separately reared in an isolator of 700 ml transparent polyethylene container filled with 500 ml of artificial seawater. The isolators allowed us to observe their defecation behavior. All seawater in the isolator was changed twice a week. Sea cucumbers were fed an artificial diet containing powdered algae and diatomaceous earth developed by Unuma et al. (2013) in an incubator at 13 °C. Feces from these specimens were collected in 1.5 ml of sterilized tubes under a portable clean bench system (Pure Space, AS ONE, Osaka, Tokyo). These were immediately stored at −80 °C before DNA extraction and purification. After sample collection defined as “time-point 0 (zero)”, the internal organs of five individuals were removed by injection of 0.35 M KCl into their body cavities using a 1.0 ml syringe and a 27G needle (Terumo, Tokyo, Japan) due to the fact mechanical removal of the gut were not possible due to the small body sizes. Sea cucumbers expelled internal organs within a minute. Gut regeneration was assessed by observation of defecation (Fig. S2); Regen-1 at 16 days after gut removals, Regen-2 at 24 days, and Regen-3, -4 and -5 at 15 day. Feces were collected from the eight specimens of sea cucumbers at ca. five time points indicated in Fig. S2 with red circles. Samples were immediately stored at −80 °C until DNA extraction.

### Microbial DNA preparation and 16S rRNA gene sequencing

After rapid thawing of the fecal samples, microbial DNA extraction and purification were performed using the NucleoSpin Soil Kit (MACHEREY-NAGEL, Düren, Germany) with a beads-beating system (Beads crusher μt-12; TAITEC Corp., Koshigaya, Japan) according to the manufacturer’s protocol.

16S rRNA gene sequencing was performed using the purified DNA samples. The hypervariable V1–V2 region of the 16S rRNA gene was amplified by PCR with 27Fmod and 338R primers containing barcode and Illumina adaptor sequences. PCR amplicons were purified using AMPure XP magnetic purification beads (Beckman Coulter, Brea, CA, USA), and quantified using the Quant-iT PicoGreen dsDNA Assay Kit (Life Technologies, Japan). Equal amounts of each PCR amplicon were mixed and then sequenced using MiSeq Reagent Kit v3 (600-cycles) on the MiSeq Illumina platform. Based on sample specific barcodes, obtained reads were assigned to each sample.

### Quality control and taxonomic assignment

The paired-end sequence data with quality scores (i.e., Fastq files) were analyzed using QIIME 2 (Bolyen et al., 2019). We performed quality controls (e.g., trimming primers and denoising sequences, removing chimeric sequences) and merged paired-end sequences using “dada2 denoise-paired” command (Callahan et al., 2016). Actually based on read quality profiling, reads trimmed 20 bp at 5′ termini and truncated at 230–280 bp positions were used for downstream analyses. As reads with 100% similarity constituted a feature (i.e., amplicon sequence variance (ASV)), each feature was taxonomically assigned using the Naive Bayes classifier implemented in the QIMME 2 package and manually trained Greengenes database version 13.8.

In the caged mariculture experiment, a total of 1,049,891 paired-reads from 29 samples from seven individuals were obtained, and 519,815 bacterial reads (2,528 features), 106,307 chloroplast reads (163 features), and 5 mitochondria reads (1 feature) were retained after quality control (Table S1). The chloroplast reads were further re-assigned to algal taxa using the Naive Bayes classifier and PhytoRef database (Decelle et al., 2015). The mitochondria reads were not used for the downstream analysis.

In the laboratory isolator experiment, a total of 2,470,596 paired-reads from 49 samples from eight individuals were obtained, and 1,767,900 bacterial reads, constituting 666 features, were retained after quality control (Table S2). All eukaryotic reads were removed due to the small number of reads (Table S2).

### Diversity analyses

Using subsampled reads (5,000 bacterial reads and 1,000 eukaryotic reads in the caged mariculture experiment, and 20,000 bacterial reads in the laboratory experiment), alpha and beta diversity analyses were performed using the QIIME2. The Shannon index as alpha diversity was compared between regenerated individuals and controls, and visualized in boxplots. Statistical tests were performed with QIIME2 plugins using the Kruskal–Wallis test. Unweighted UniFrac distances as beta diversity were calculated and visualized in PCoA plots (Lozupone et al., 2011) based on the phylogenic tree generated by FastTree. Statistical tests were performed with QIIME2 plugins using permutational multivariate analysis of variance (PERMANOVA). Results were considered to be statistically significant with a false discovery rate (FDR)-adjusted *p*-value < 0.05.

### Relative abundance comparison

In the laboratory isolator experiment, features composed of over 500 reads were retained. We calculated differences in relative abundance of the features between time-point 0 and time-point 17 in each specimen, and visualized the differences using heat maps of the features overlaid on the phylogenetic tree reconstructed using FastTree. Specimen “Regen-2” was excluded in this analysis due to the limited number of samples. Key features were defined as features in which the change in relative abundance from time-point 0 to time-point 17 were over 3.0% in at least two individuals from the regeneration group.

Temporal changes of relative abundance of key features were also analyzed in a line plot. Welch’s t test was performed to compare the relative abundance of the features between regenerated and control individuals at each time-point. Results were considered to be statistically significant with a *p*-value < 0.05.

### Isolation of sea cucumber microbiota

Sea cucumber (*A. japonicus*) samples including eggs, larvae, and feces from regenerated guts were used to isolate bacteria of interest. Eggs or larvae were homogenized in sterilized seawater for 1 min, and then ten-fold dilutions of the homogenates were made in sterilized seawater. A total of 10 μl of the 10^−2^ or 10^−3^ dilution was spread on a modified ZoBell 2216E agar plate (polypeptone 0.5%, yeast extract 0.1%, agar 1.5% in 75% natural seawater). The plates were incubated at 18 °C for a week. Feces from regenerated guts were suspended in sterilized seawater, and then ten-fold dilutions of the suspension were performed in sterilized seawater. A total of 10 μl of the 10^−1^, 10^−2^ or 10^−3^ dilution was spread on the modified ZoBell 2216E agar media. The plates were incubated at 20 °C.

## Results

### Gut microbiome regeneration in natural environments

**Figure 1:**
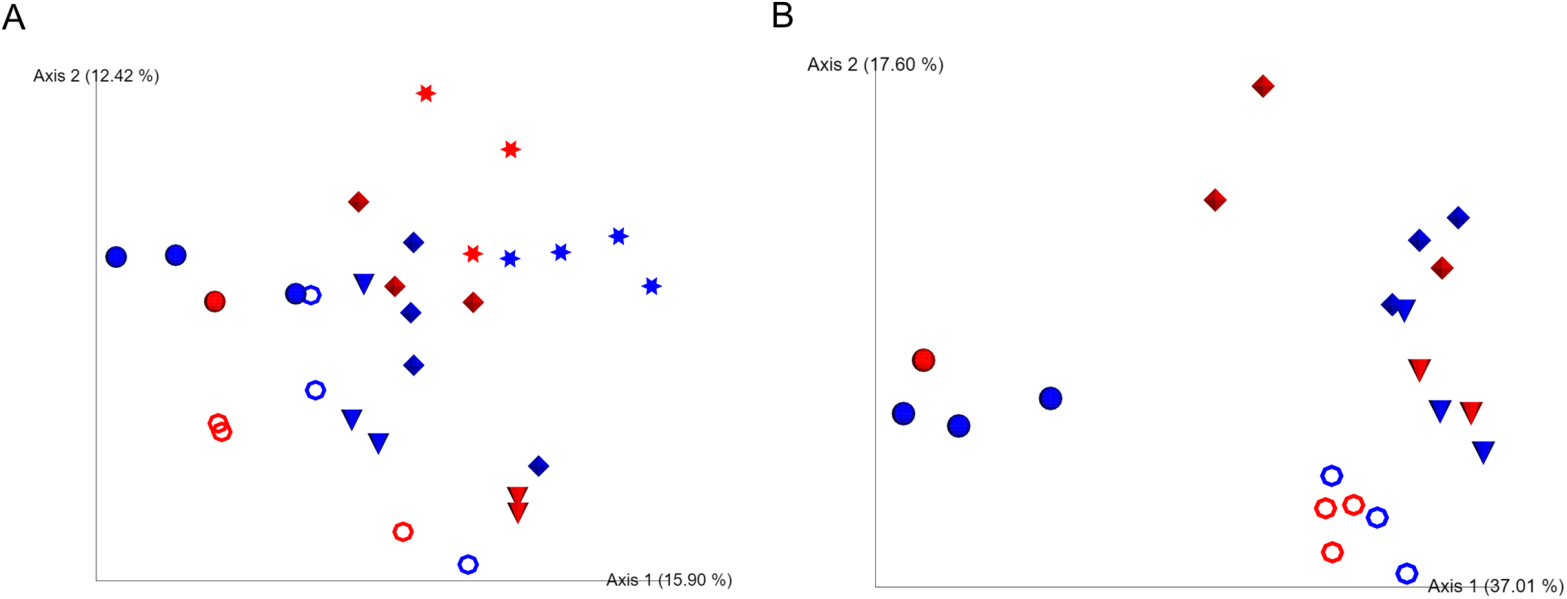
Unweighted UniFrac analyses in the caged mariculture experiment. Red and blue colored symbols show gut-regenerated and control groups, respectively. Star-shaped symbols represent samples collected in December, when the gut of sea cucumber was removed. Closed circle, open circle, cone, and diamond-shaped symbols represent samples collected in April, May, June, and July, respectively. (A) Bacterial communities, and (B) eukaryotic communities. December samples were excluded due to limited number of eukaryotic reads.

We were unable to start observation of sea cucumber feeding status and collections of fecal samples until 4 months after evisceration due to severe weather conditions in the winter in 2016 in Hokkaido, Japan. At this time point, most of the individuals with guts removed had resumed ingestion of sea bottom sediments and excreted feces in cages. Based on the Shannon index and unweighted UniFrac analyses, bacterial communities of the gut-regenerated group after 4 months of gut removal were not significantly different from those of the control group at any of the sampling dates (*p* ≥ 0.05) (Fig. 1A; Fig. S3). Gut eukaryotic communities were also not significantly different between the regenerated and control groups (*p* ≥ 0.05) (Fig. 1B).

### Early-stage dynamics of gut microbiome using the laboratory isolator

**Figure 2:**
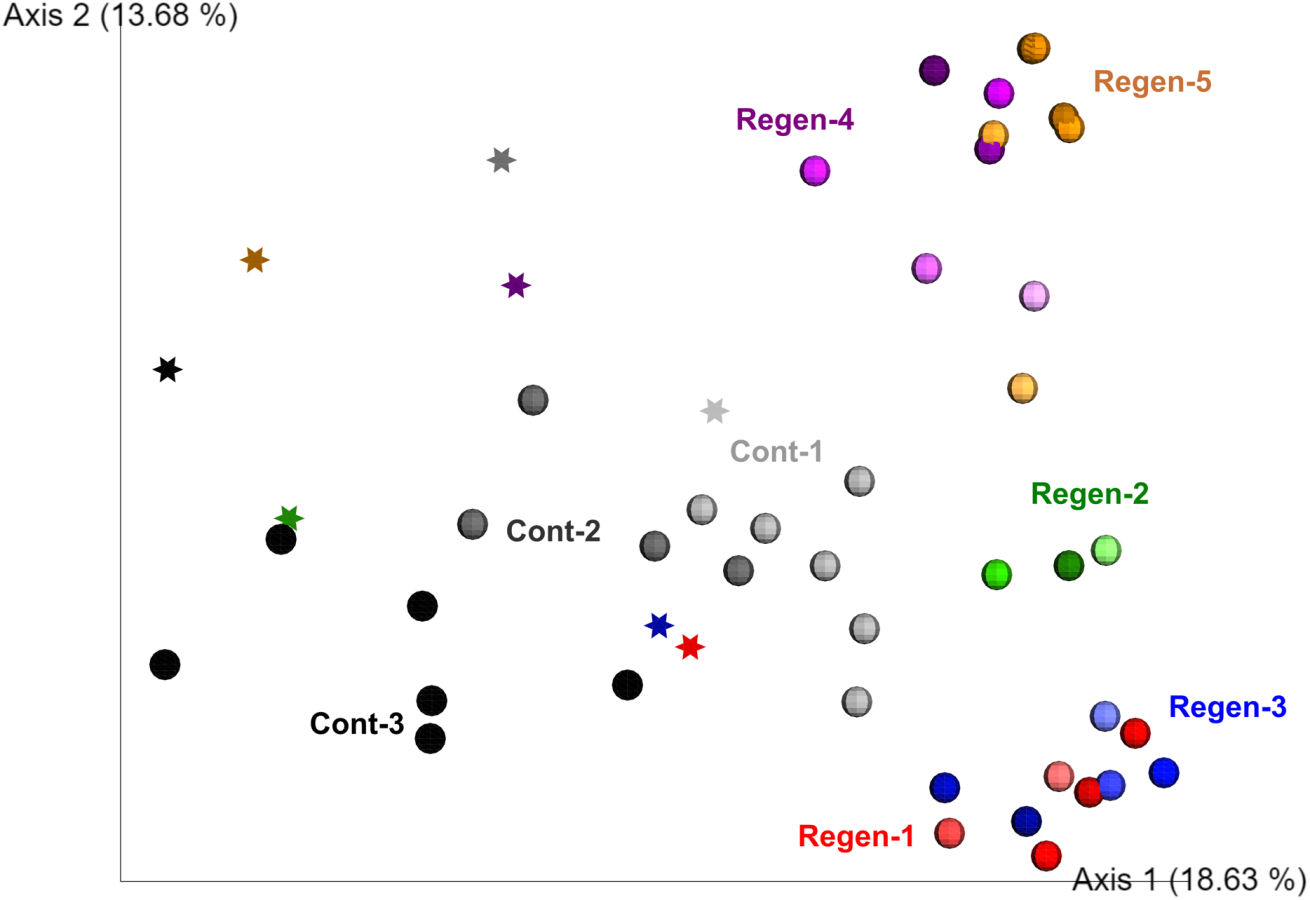
Unweighted UniFrac distance-based 2D PCoA in the laboratory isolator. Specimens in the regeneration group are indicated by “Regen-” with red, green, blue, purple, and orange circle depend on individual. In the gut-regenerated individual, dark to shaded gradation in each color symbol indicates temporal scale after gut-regeneration. Specimens in the control group are indicated by “Cont-” with gray scale. Star indicates samples collected before evisceration.

To fill gaps in the understanding of early-stage dynamics of gut microbiome along with gut regeneration, we set up a laboratory isolator to monitor the dynamics of the fecal microbiota derived from identical specimens for 1 month and compared the regeneration group (five individuals) and the control group (three individuals) (Fig. S2). Alpha diversity of gut-regenerated individuals was likely to be higher than those in the control group, but it was not significantly different at any time points after evisceration (*p* ≥ 0.05) (Fig. S4). Moreover, PERMANOVA based on unweighted UniFrac disitances indicated that bacterial communities were significantly different between specimens (*p* < 0.01), and fecal microbiota collected before evisceration appeared to be different from those collected after evisceration (*p* < 0.01) (Fig. 2).

**Figure 3:**
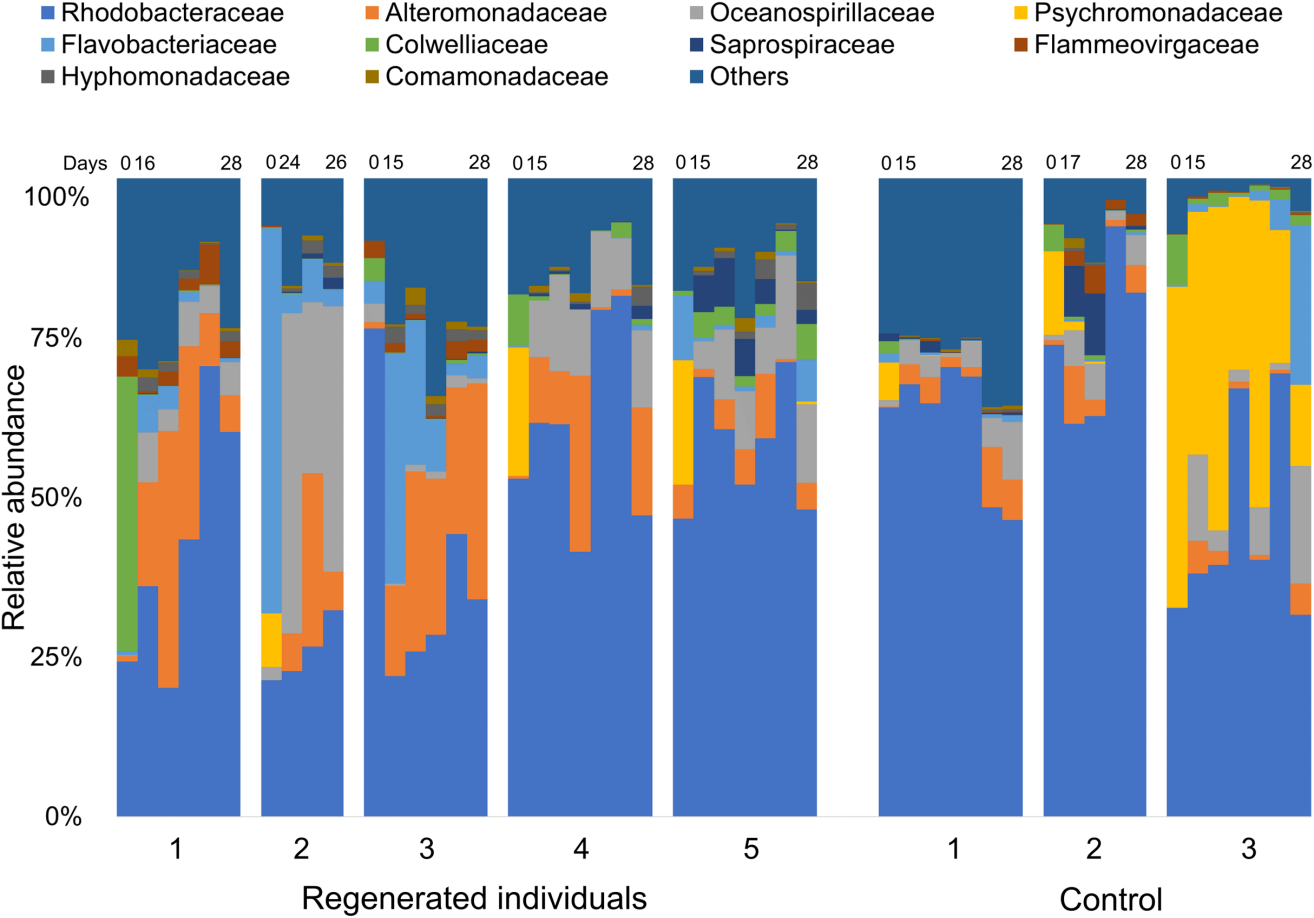
Bacterial community dynamics at family level in the laboratory isolator. The top 10 families are shown in the bar plot. Minor taxa and unassigned reads are clustered into others. Upper numbers are time points corresponding to Fig. S2.

Comparison of the fecal bacterial composition by specimen at family level also showed inter-individual variation, although Rhodobacteraceae was the most abundant family in most specimens, followed by Alteromonadaceae, Flavobacteriaceae, and Oceanospirillaceae (Fig. 3). In addition, Colwelliaceae accounted for 43.1% of fecal microbiota of “Regen-1” at time-point 0, but this family decreased below 1% after gut regeneration (Fig. 3). Similarly, Flavobacteriaceae and Rhodobacteraceae in specimens “Regen-2 and -3”, respectively, decreased after gut regeneration. However, fecal microbiota of the control group did not change dramatically (Fig. 3).

**Figure 4:**
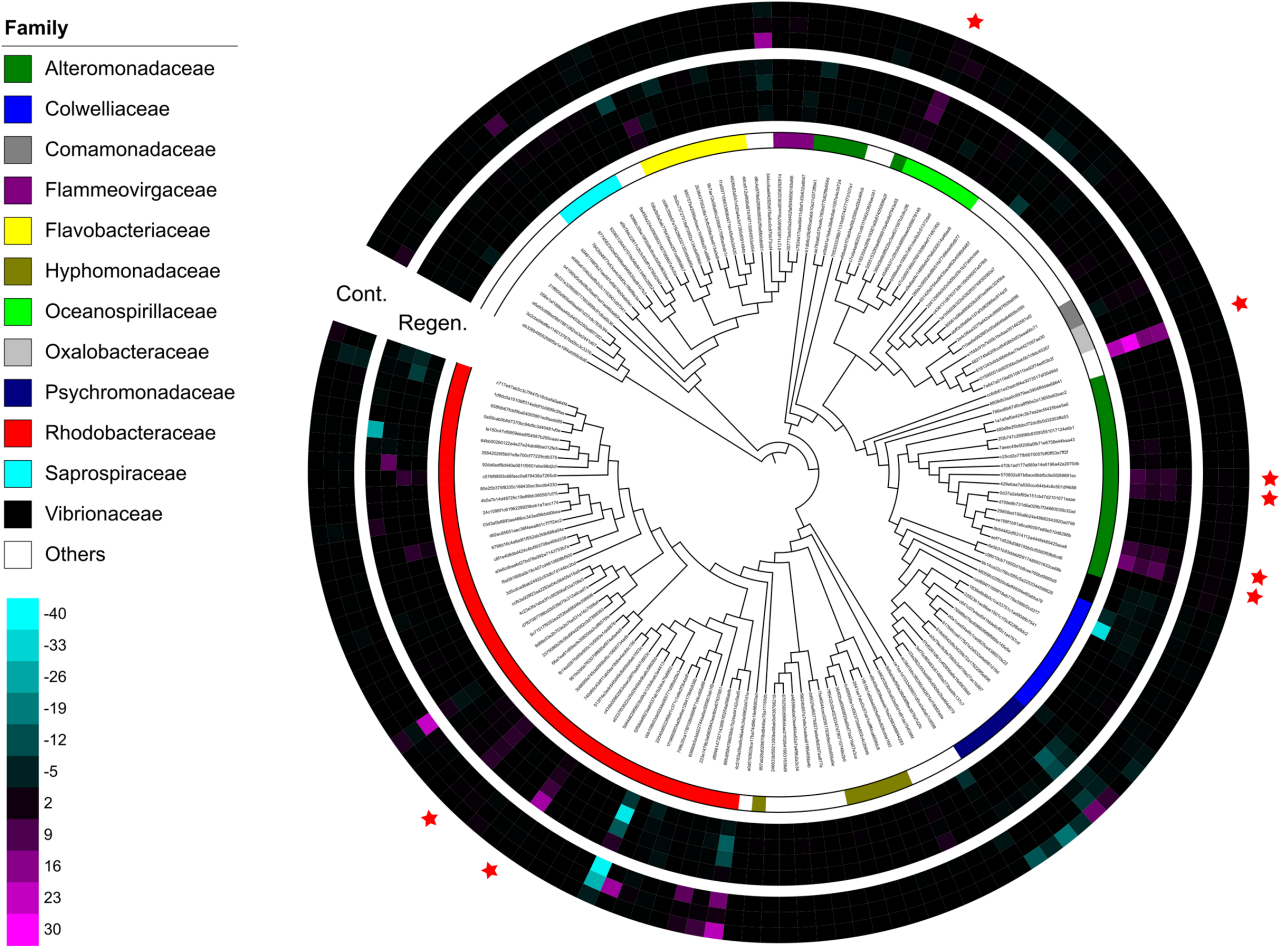
Dendrogram-connected heatmaps features in the laboratory isolator. Difference of relative abundance of features with >500 reads between time-point zero and time-point 17 was calculated. The heat maps show four individuals from the regeneration group and three individuals from the control group, and specimen “Regen-2” was excluded due to limited number of samples. More vivid magenta corresponds to more abundant OTUs in time-point 17 than in time-point 0, and more vivid cyan corresponds to more abundant OTUs in time-point 0 than in time-point 17. Features are placed on a maximum-likelihood tree on the basis of representative reads of each feature. The color bar shows family level affiliation of each feature. Unassigned and minor taxa are combined into others. Red stars indicate key features whose relative abundance increased after evisceration in the regeneration group.

**Figure 5:**
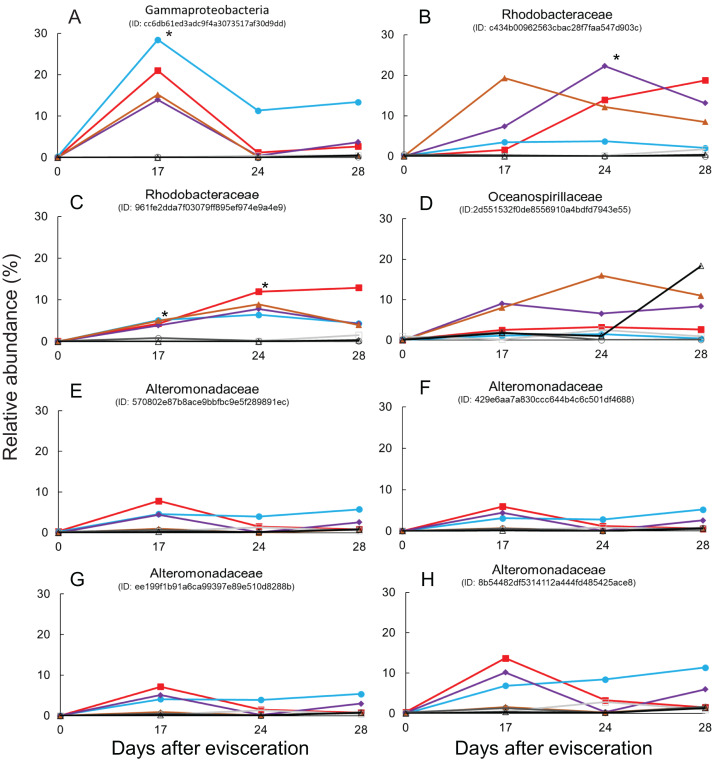
Dynamics of relative abundance of key features. Eight key features are shown in (A–H), respectively. * indicates statistically significant (*p* < 0.05) compared to control group. Gut-regenerated individuals no. 1, 3, 4 and 5 are indicated by red-closed square, aqua blue-closed circle, purple-closed diamond, and brown-closed triangle, respectively. Control group no. 1 to 3 are indicated by light-gray open square, gray open circle, and black open triangle, respectively.

Immediate increases in the relative abundance of eight key features were observed after gut regeneration (time-point 17), and notably, little differences between time-point 0 and 17 were shown in the control group (Fig. 4). One feature was assigned to Gammaproteobacteria (order unknown) showing 98% similarity to an uncultured clone from a coral black band disease bacterial community (Ben-Dov et al., 2011) (Fig. 5A), two features to Rhodobacteraceae including *Sulfitobacter pontiacus* (100% similarity) (Figs. 5B and 5C), one to Oceanospirillaceae (Fig. 5D), and four features to Alteromonadaceae (100% similarity to *Alteromonas stellipolaris*) (Figs. 5E and 5F). These eight key features can be further divided into at least two types based on relative abundance dynamics; (1) abundance maximum at time-point 17 day (Figs. 5A and 5E–5H), and (2) that at time-point 24 day (Figs. 5B–5D). In the first type, the abundance dramatically increases at time-point 17, but those were likely to return back to the baseline level at later stages of gut regeneration (Figs. 5A and 5E–5H), in particular, significant increases were observed in the feature shown in Fig. 5A. Moreover, all four Alteromondaceae features were affiliated to *A. stellipolaris* strains. The sum of relative abundance of the *A. stellipolaris* features (20.4 ± 12.7) were significantly abundant at the 17th day in the gut-regenerated group comparing to that of control group (1.85 ± 1.25) (Welch T, *p* = 0.0345) (Figs. 5E–5H). On the other hand, in the second type, when the relative abundance maximum reached at time-point 24, and even at time-point 28, abundance did not return to the baseline (Fig. 5C).

### Isolation of key features

Comparison of 16S rRNA gene sequences between eight key features and isolates using a basic local alignment search tool (BLAST) demonstrated that isolates related to the above six features showing >98.7% similarity were found. The partial 16S rRNA sequence of the strain BLE2, which was isolated from blastula larvae and identified as *A. stellipolaris* (Alteromonadaceae), were similar to the sequences from four features (Figs. 5E–5H). One feature (Fig. 5C) was similar to the strain BL28, which was isolated from blastula larvae and identified as *S. pontiacus* (Rhodobacteraceae). The other feature (Fig. 5D) was similar to the strain PT12, which was isolated from pentactula larvae and identified as *Neptunomonas phycophila* (Oceanospirillaceae).

## Discussion

Individuality of gut microbiota, whose species composition and relative proportions of dominant microbial groups vary tremendously, has been demonstrated in vertebrates, including humans associated with host and environmental factors. Even when constantly being exposed to environmental challenges, its composition and function in an individual is stable against perturbations (Benson et al., 2010; Sommer et al., 2017). Aquatic animals are more likely to live in water environments involving multiple perturbation factors compared to those of humans, however, resilience of gut microbiota at individual level on a fine temporal scale has not been fully tested yet due to a lack of methodology. Using individual microbiome methodology (Yamazaki et al., 2016, 2019, 2020) and a gut-regenerating animal model of sea cucumber *A. japonicus*, which is located on a deep branch of Deuterostomia, we succeeded in tracking the individuality of the gut microbiome in marine invertebrate animals during the organ regeneration process under both open water and laboratory conditions.

Firstly, we tested whether resilience of gut microbiomes of sea cucumber can be observed tracking individual gut microbiome dynamics during gut regeneration using caged wild animals. At least after 4 months, which is likely to be enough time to complete gut regeneration of tested animals even in winter, based on unweighted UniFrac analysis, both fecal bacterial and eukaryotic communities of the gut-regenerated group were not significantly different from those of the control group at the same sampling date (Fig. 1A; Fig. S3). Overall fecal bacterial communities between the two groups were also not significantly different, which indicate that normal bacterial communities are reconstructed after these periods after evisceration even in natural open water conditions and without overlapping individual behaviors. The mariculture experimental setup not only supports previous studies on the resilience of gut microbiomes observed in *A. japonicus* and *S. briareus* (Wang et al., 2018; Zhang et al., 2019; Weigel, 2020) but also suggest such resilience were conserved individually. Gut eukaryotic communities of the gut-regenerated individuals were not significantly different from those of the control group (Fig. 1B), which indicates that feeding behaviors of sea cucumbers became active at that time. Such short-time reconstruction of the gut microbiomes might suggest immediate recovery of holobiont functions which benefit the host via microbial metabolisms such as degradation and mineralization of ingested algae and organic matter (Gao et al., 2014; Yamazaki et al., 2019; 2020). Unfortunately, we could not perform fine temporal scale sampling using wild animals at that time in 2016 due to the severe winter ocean conditions around this area. We will be able to set up future mariculture experiments to monitor the daily dynamics if a better sea cucumber biologging system is developed.

Secondly, we tested individuality under a laboratory set up using an isolator. As the gut microbiome of *A. japonicus* is shaped by ingested sedimentary materials (Yamazaki et al., 2019), to avoid cross-contamination of the other individual microbiomes, we set up a laboratory isolator to feed individual animals and monitor daily gut microbiome dynamics. Before starting the experiments, we expected the individual fecal microbiota at the same regeneration stage would be grouped to each other as reported by Zhang et al. (2019), but our study using isolators did not show such trends during the 28 day regeneration experiments (Fig. 2). Our experimental set up was only performed within a month, so the community was in the process of reverting being back to its original microbial communities (Wang et al., 2018), but, interestingly, the microbiome regeneration process at individual level seemed to be significantly different (Figs. 2 and 3). During the regeneration process, it was reported that alpha diversity including the Shannon index dropped in the early regeneration stage but increased in the later stages (Wang et al., 2018; Zhang et al., 2019), but no significant differences in the Shannon index were observed in this study at individual level (Fig. S4). We suggest that individuality of gut microbiomes observed in sea cucumber in this study might be an evolutionary conserved biological system in Deuterostomia species.

Finally, some unique bacterial strains related to the gut regeneration were isolated. The relative abundance of Rhodobacterales was shown to significantly increase in gut microbiota of *A. japonicus* 14 and 21 days after evisceration in a previous study (Zhang et al., 2019). Rhodobacteraceae (order Rhodobacterales) is one of the key components of sea cucumber gut microbiota based on previous results; (1) Rhodobacterales bacteria were also enriched in sea cucumber guts (Yamazaki et al., 2019); (2) they were more abundant in larger size individuals than in smaller size ones (Yamazaki et al., 2016); (3) potential probiotics, *Paracoccus marcusii*, have been reported based on their effects on host growth and immune stimulation after treatment with diets (Yan et al., 2014; Yang et al., 2015). Interestingly, two key features affiliated to Rhodobacteraceae responding to gut regeneration were extracted; one was related to an uncultured clone, of which the roles are currently difficult to be estimated (Sunagawa et al., 2009), but the other was likely to be affiliated to *S. pontiacus*. *S. pontiacus* was isolated from seawater of H_2_S–O_2_ interface of the Black Sea, and the strain was characterized as possessing high sulfite oxidation activity (Sorokin, 1995). The species also produces PHB-like granules (Sorokin, 1995). The *S. pontiacus*-related feature showed unique dynamics in abundance, gradually increasing depending on gut regeneration, reaching a peak at 24 days after evisceration, and remaining at the 28 day stage in most of the gut-regenerated individuals, and therefore seems to be a healing resident (Fig. 5C). The species could be a probiotics candidate in supporting gut regeneration of sea cucumber *A. japonicus*. Fortunately, we also successfully isolated the *S. pontiacus* related strain BL28 from a gastrula larvae of the sea cucumber *A. japonicus*. We did not have growth rate information (e.g., body weight and length) during this isolator experiments because to prevent stressing individuals we avoided handling them in this experiment, but the isolator could function as a superior bioassay set up. A bioassay measuring correlations between host growth and gut microbiome dynamics could help further studies in discovering beneficial microbes in gut regeneration.

Alteromonadaceae is also abundant in the gut of sea cucumber *A. japonicus*. Interestingly, significant abundant maximum of *A. stellipolaris* features was observed at the 17 day stage in all gut-regenated animals (Figs. 5E–5H). Recently, complete genome analyses of *A. stellipolaris* revealed genes responsible to quorum quenching (QQ) activity (Torres et al., 2019). As quorum sensing (QS) regulates a wide variety of gene expression responsible for biofilm formation, virulence, the QQ activity during gut regeneration might play a role in preventing specific pathogens and colonizers colonizing on regenerated gut tissues, which could help the recovering holobiont. These bacteria are newly listed as probiotics candidates according to this study.

## Conclusions

We developed both caged mariculture and laboratory isolator systems of sea cucumbers to track dynamics of the gut metagenomes derived from the same specific individuals. In the caged mariculture experiment, we confirmed that bacterial and eukaryotic communities of sea cucumbers’ guts are reconstructed within 4 months of evisceration. The laboratory isolator experiment indicated that eight key bacteria belonging to Alteromonadaceae, Rhodobacteraceae, Oceanospirillaceae and family unassigned Gammaproteobacteria may be related to the gut regeneration process. Using the developed individual gut microbiome tracking, both individuality and resilience of gut microbiome of sea cucumber were described, which might be shared traits in Deutrostomia species. We also isolated strains which are related to the key bacteria, the effects on gut regeneration will be tested in the future.

## Supplemental Information

10.7717/peerj.10260/supp-1 Supplemental Information 1 Supplemental Materials.

## Additional Information and Declarations

### DNA Deposition

The following information was supplied regarding the deposition of DNA sequences: These data are also available at Figshare: Sawabe (2020): Raw_reads.zip. figshare. Dataset. DOI 10.6084/m9.figshare.12043305.v1.

### Data Availability

The following information was supplied regarding data availability: All sequences are available in GenBank: DRR231760–DRR231820.
