# Supplementary material for: Tracking the dynamics of individual gut microbiome of sea cucumber Apostichopus japonicus during gut regeneration
Source: PeerJ. 2020 Dec 1;8:e10260. doi: 10.7717/peerj.10260 (PMC7718794; doi:10.7717/peerj.10260)
Supplement: Supplemental Information 1 [file peerj-08-10260-s001.pdf]

Table S1. Sample information in caged mariculture experiment

sample-id	specimen-id	Month	Regeneration	No. of raw reads	No. of qualified bacterial reads	No. of qualified chloroplast reads	No. of qualified mitochondria reads
ApF17	Cont-B	Apr	Control	23,501	5,859	9,461	0
ApF41	Cont-C	Apr	Control	26,915	6,463	11,577	0
ApF42	Regen-C	Apr	Regeneration	28,094	10,621	8,369	0
ApF51	Cont-C	Apr	Control	28,261	9,178	9,532	0
DF101	Cont-A	Dec	Control	30,207	16,717	1,847	0
DF102	Cont-C	Dec	Control	46,624	25,886	2,334	0
DF103	Cont-D	Dec	Control	36,146	20,189	2,184	0
DF16	Regen-A	Dec	Regeneration	25,152	15,012	499	0
DF17-170126	Cont-B	Dec	Control	33,116	16,720	1,117	0
DF18	Regen-B	Dec	Regeneration	24,115	14,450	819	0
DF42	Regen-C	Dec	Regeneration	26,471	15,628	342	0
JulyF15	Cont-A	July	Control	47,288	24,818	1,591	0
JulyF16	Regen-A	July	Regeneration	44,579	23,472	1,722	0
JulyF17	Cont-B	July	Control	41,111	21,876	723	0
JulyF18	Regen-B	July	Regeneration	41,880	21,375	1,369	0
JulyF41	Cont-C	July	Control	50,814	24,298	1,550	0
JulyF42	Regen-C	July	Regeneration	46,035	22,834	1,971	0
JulyF51	Cont-D	July	Control	40,304	21,342	1,816	0
JunF15	Cont-A	Jun	Control	35,627	19,486	2,311	0
JunF17	Cont-B	Jun	Control	30,872	16,565	1,301	0
JunF18	Regen-B	Jun	Regeneration	56,386	27,927	5,483	0
JunF42	Regen-C	Jun	Regeneration	60,535	33,770	2,534	0
JunF51	Cont-D	Jun	Control	29,306	16,520	1,817	0
MaF15	Cont-A	May	Control	48,389	17,776	9,745	0
MaF16	Regen-A	May	Regeneration	32,013	16,264	4,837	0
MaF18	Regen-B	May	Regeneration	27,195	13,164	3,604	0
MaF41	Cont-C	May	Control	32,090	15,751	5,413	0
MaF42	Regen-C	May	Regeneration	32,194	16,667	3,582	0
MaF51	Cont-D	May	Control	24,671	9,187	6,857	5

Table S2. Sample information in the laboratory feeding experiment

sample-id	Specimen-id	Group	Time-points	No. of raw reads	No. of qualified bacterial reads	No. of qualified eukaryotic reads
A10May19	Regen-1	Regeneraion	18	29,501	21,959	89
A16May19	Regen-1	Regeneraion	24	49,278	37,805	51
A20May19	Regen-1	Regeneraion	28	70,620	52,970	28
A22Apr19	Regen-1	Regeneraion	0	32,553	24,433	113
A8May19	Regen-1	Regeneraion	16	46,632	35,733	22
A9May19	Regen-1	Regeneraion	17	32,531	24,962	51
B16May19	Regen-2	Regeneraion	24	121,076	81,388	0
B17May19	Regen-2	Regeneraion	25	53,693	36,288	35
B18May19	Regen-2	Regeneraion	26	54,440	43,282	16
B22Apr19	Regen-2	Regeneraion	0	34,556	27,402	0
C16May19	Regen-3	Regeneraion	24	58,718	42,642	237
C20May19	Regen-3	Regeneraion	28	52,457	41,050	25
C22Apr19	Regen-3	Regeneraion	0	33,162	25,337	15
C7May19	Regen-3	Regeneraion	15	34,141	25,887	56
C8May19	Regen-3	Regeneraion	16	36,794	27,751	27
C9May19	Regen-3	Regeneraion	17	39,881	29,382	20
D12May19	Cont-1	Control	20	50,097	35,312	0
D16May19	Cont-1	Control	24	54,991	40,947	3
D20May19	Cont-1	Control	28	62,294	46,189	0
D22Apr19	Cont-1	Control	0	30,327	23,749	44
D7May19	Cont-1	Control	15	39,352	28,417	0
D8May19	Cont-1	Control	16	36,690	24,377	0
D9May19	Cont-1	Control	17	41,361	26,517	0
E12May19	Regen-4	Regeneraion	20	63,538	43,934	8
E16May19	Regen-4	Regeneraion	24	66,153	45,770	17
E20May19	Regen-4	Regeneraion	28	60,521	44,656	74
E22Apr19	Regen-4	Regeneraion	0	39,301	28,965	0
E7May19	Regen-4	Regeneraion	15	31,806	23,629	173
E8May19	Regen-4	Regeneraion	16	42,856	28,268	50
E9May19	Regen-4	Regeneraion	17	66,034	42,544	355
F12May19	Regen-5	Regeneraion	20	60,851	43,100	10
F16May19	Regen-5	Regeneraion	24	60,068	42,283	9
F20May19	Regen-5	Regeneraion	28	61,939	45,378	13
F22Apr19	Regen-5	Regeneraion	0	44,891	34,352	3
F7May19	Regen-5	Regeneraion	15	40,297	28,828	277
F8May19	Regen-5	Regeneraion	16	58,208	40,157	44
F9May19	Regen-5	Regeneraion	17	38,815	25,338	39
G12May19	Cont-2	Control	20	76,418	45,891	6
G16May19	Cont-2	Control	24	92,206	61,219	2
G20May19	Cont-2	Control	28	58,701	42,755	0
G22Apr19	Cont-2	Control	0	35,838	25,284	17
G9May19	Cont-2	Control	17	64,161	41,145	48
H12May19	Cont-3	Control	20	52,204	39,918	0
H16May19	Cont-3	Control	24	59,479	45,594	0
H20May19	Cont-3	Control	28	58,916	44,376	0
H22Apr19	Cont-3	Control	0	31,463	22,828	0
H7May19	Cont-3	Control	15	35,452	26,964	0
H8May19	Cont-3	Control	16	37,861	26,525	0
H9May19	Cont-3	Control	17	37,474	24,420	0

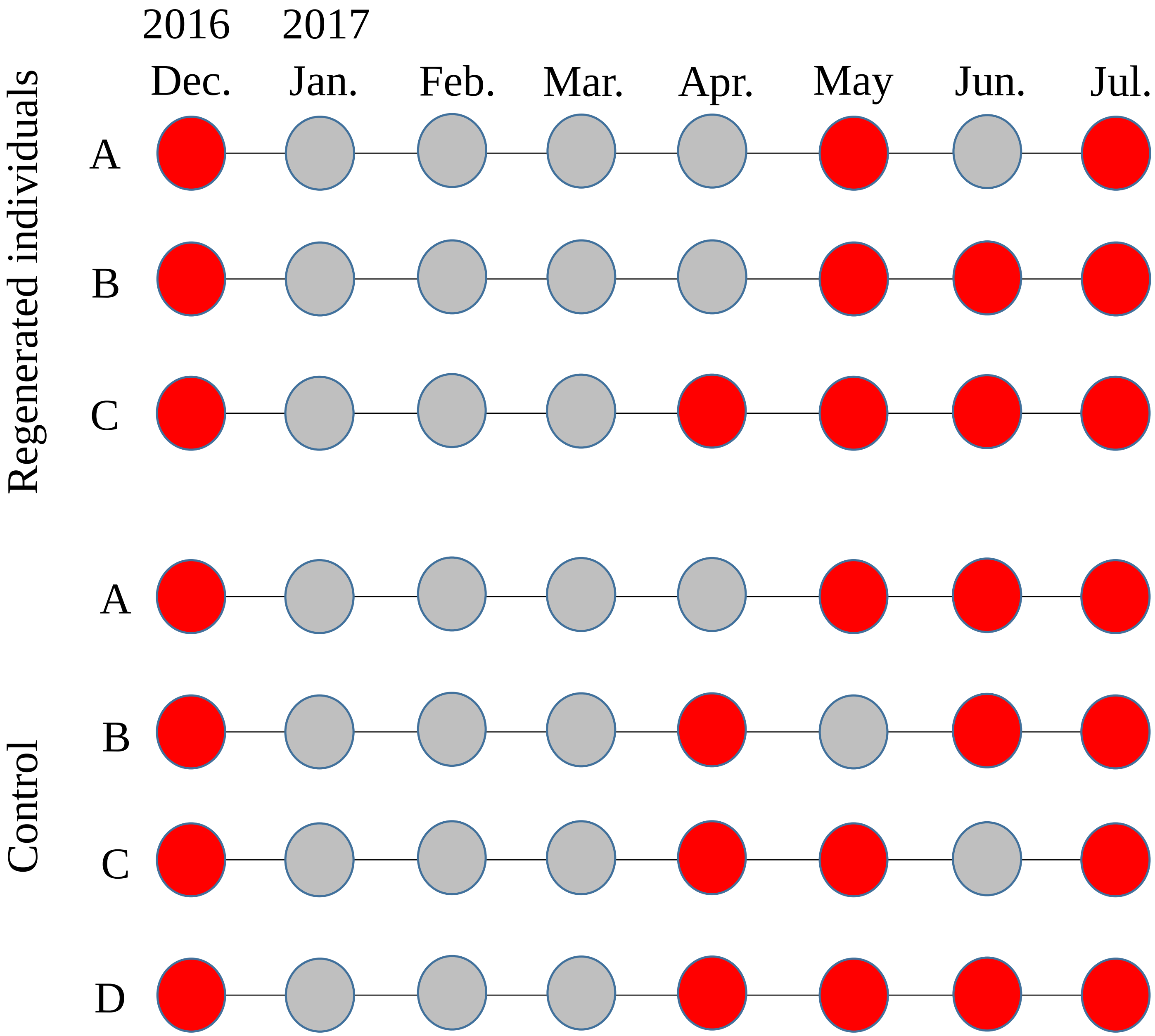

Fig. S1. Experimental design of the caged mariculture experiment. Fecal samples were collected in time points with marked red circles. I removed sea cucumbers' guts on December 2016. Their guts were already regenerated in April 2017.

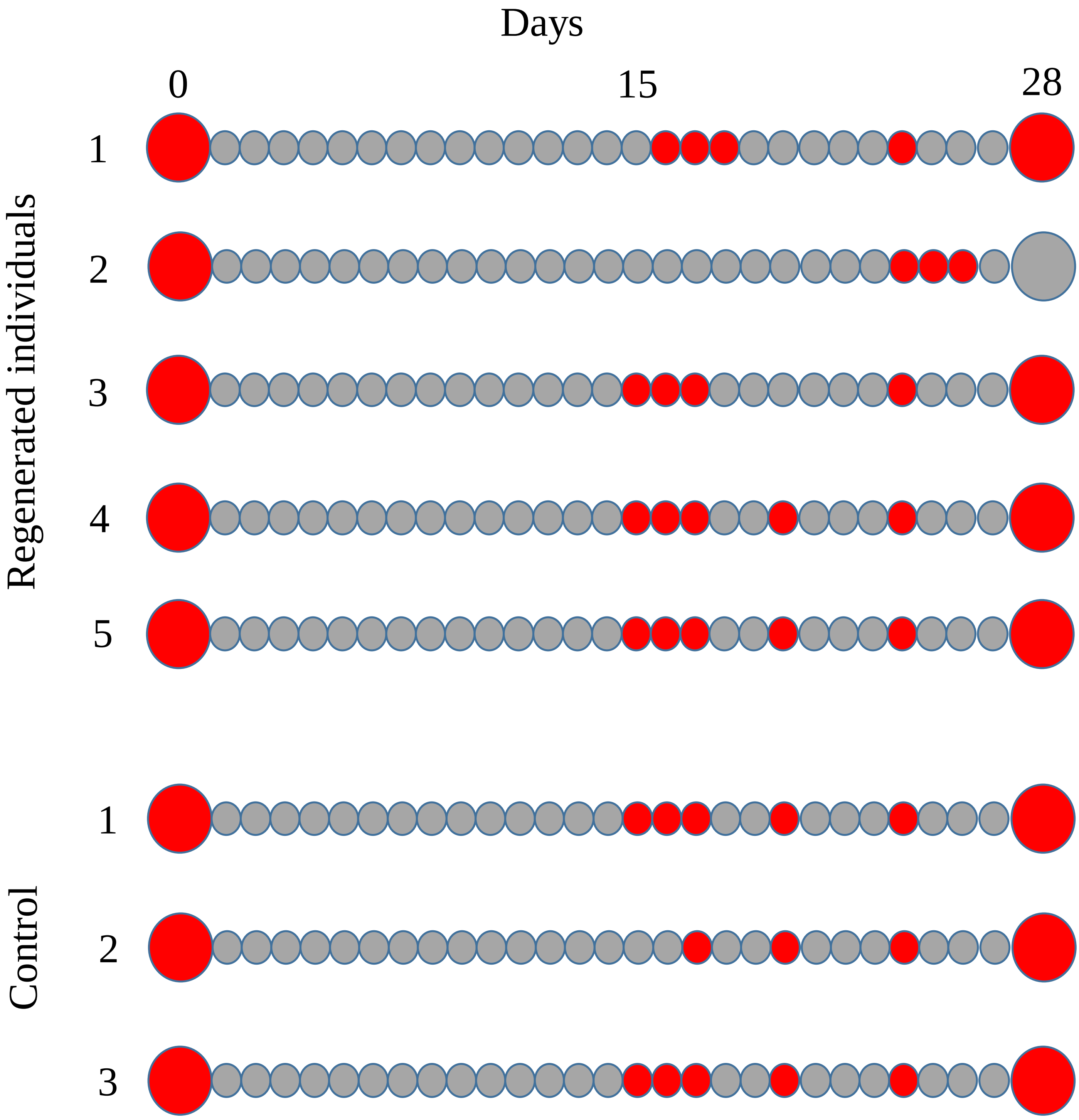

Fig. S2. Experimental design of the laboratory feeding experiment. Fecal samples were collected in time points with marked red circles. I removed sea cucumbers' guts at time-point 0. Eviscerated guts were regenerated in 15 days (specimen-ID; Regen-3, -4 and -5), 16 days (Regen-1) or 24 days (Regen-2) after gut removal.

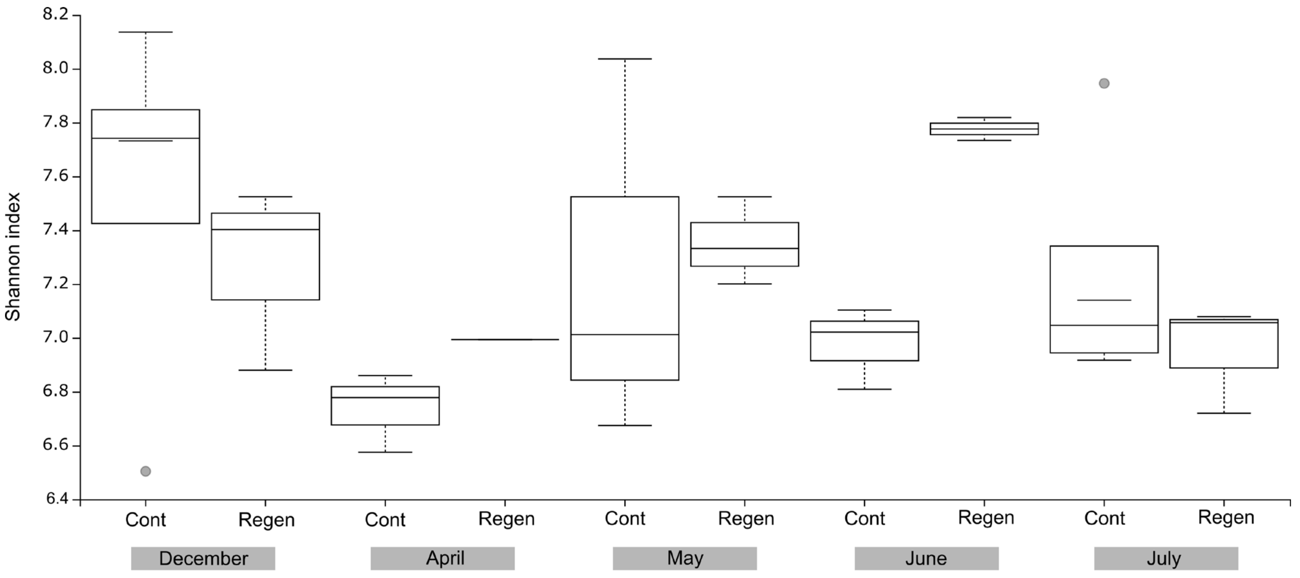

Fig. S3. Shannon index comparisons between the regeneration group and controls in the mariculture experiment. The regeneration group is indicated by "Regen.", and the control group is indicated by "Cont.". (A) Samples collected in December 2016 (i.e. pre-evisceration) were analyzed. (B) Samples collected in April, May June and July 2017 (post-evisceration) were analyzed. N.S. stands for not significant (*p*>=0.05).

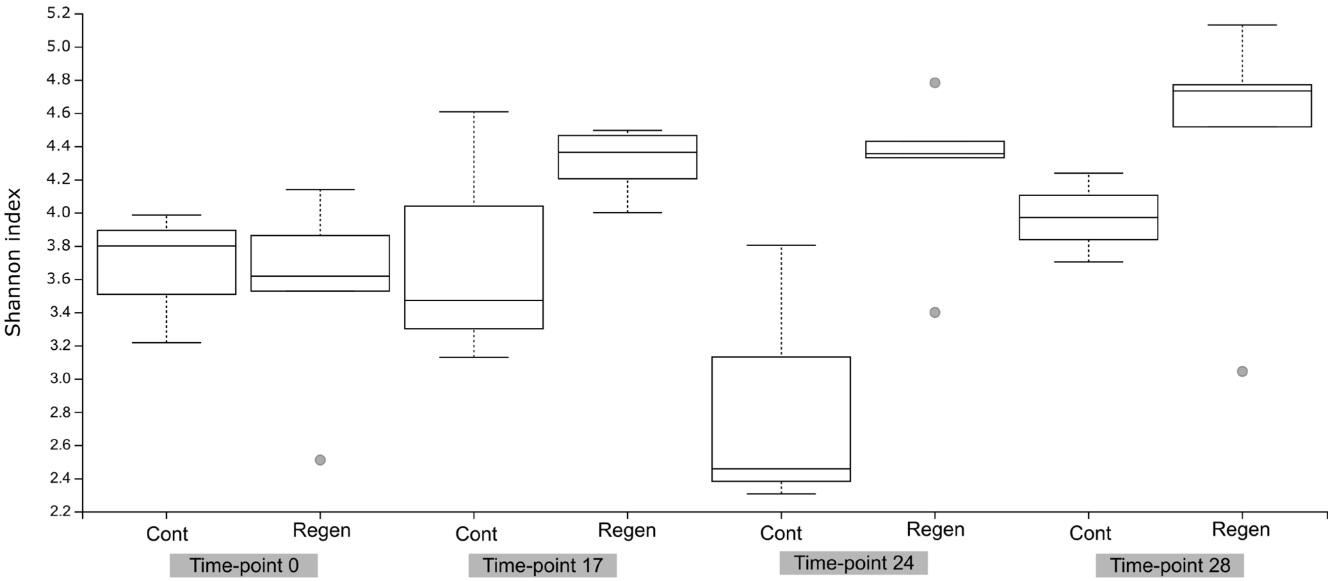

Fig. S4. The Shannon index comparisons between the regeneration group and controls in the laboratory isolator. The regeneration group is indicated by “Regen.”, and the control group is indicated by “Cont.”. (A) Samples collected at time-point 0 were analyzed. N.S. stands for not significant (p>=0.05). (B) Samples collected at all time-points of post-evisceration were analyzed. N.S. stands for not significant (p>=0.05).
